# Highly Sensitive Detection of Clenbuterol in Animal Urine Using Immunomagnetic Bead Treatment and Surface-Enhanced Raman Spectroscopy

**DOI:** 10.1038/srep32637

**Published:** 2016-09-07

**Authors:** Jie Cheng, Xiao-Ou Su, Shi Wang, Yiping Zhao

**Affiliations:** 1 Institute of Quality Standards and Testing Technologies for Agro-products, Chinese Academy of Agricultural Sciences, Beijing, 100081, China; 2 Department of Physics and Astronomy, University of Georgia, Athens, Georgia 30602, USA

## Abstract

Combining surface-enhanced Raman spectroscopy (SERS) of aggregated graphene oxide/gold nanoparticle hybrids with immunomagnetic bead sample preparation method, a highly sensitive strategy to determine the clenbuterol content in animal urine was developed. Based on a linear calibration curve of the SERS characteristic peak intensity of clenbuterol at Δ*v* = 1474 cm^−1^ *versus* the spiked clenbuterol concentration in the range of 0.5–20 ng·mL^−1^, the quantity of clenbuterol in real animal urine samples can be determined and matches well with those determined by LC-MS/MS, while the detection time is significantly reduced to 15 min/sample. The limits of detection and quantification in the urine are 0.5 ng·mL^−1^ and 1 ng·mL^−1^, respectively, and the recovery clenbuterol rates are 82.8–92.4% with coefficients of variation <9.4%. The day-to-day variation of the detection is less than 6.41%, and the shelving life of the SERS substrates is no less than 4 weeks. All these indicate that this proposed SERS detection protocol for clenbuterol is reproducible, reliable, and can be easily developed for the routine monitoring of the illicit use of clenbuterol in animal farming.

Clenbuterol is a kind of β_2_-adrenergic agonist which is used to adjust bronchiectasis and smooth muscle relaxation^1^. It has also been used to produce leaner meat with a higher muscle to fat ratio for livestock, and is often called “lean meat powder” in China^2^. It is prohibited for use in food producing animals in both USA, European Union^3^, and China^4^. Even with strict regulations, cases of illegal use of clenbuterol still emerged, such as the Shenzhen snake-clenbuterol event in 2010^5^, the Shuanghui “lean meat powder” event in 2011^6^, and the β-agonists events reported in 2012^7^. The residues of clenbuterol remained in meat tissues consumed by a person may cause increased heart rate, muscular tremors, headaches, nausea, fever and chills. Generally, the main method to monitor the illegal use of clenbuterol in animal farming is to detect the residue of the drug from animal urine using analytical methods such as gas chromatography/mass spectrometry (GC/MS)^8^ and liquid chromatography–tandem mass spectrometry (LC-MS/MS)^9^. Though very effective and accurate, the above mentioned methods require complicated sample pretreatment steps such as solid phase extraction (SPE) clean-up and derivatization reaction operation, which all need expensive instruments and demand experienced personal.

Recently, many rapid screening methods have been proposed for clenbuterol detection, such as enzyme-linked immunosorbent assay technique^10^,^11^, immunochromatographic assay based on up-conversion phosphors^12^, fluorescent multi-component immuno chromatography^13^ and surface enhanced Raman spectroscopy (SERS)^14^,^15^. SERS is an emerging and powerful spectroscopic sensing technique based on the enhanced local electromagnetic field near the nanostructured noble metal surfaces^16^,^17^. Compared to conventional Raman spectroscopy, SERS can enhance the normal Raman signal up to 10^6^–10^12^ times, and sometime can even reach a signal molecule sensitivity^18^,^19^. However, there are few reports in literature addressing clenbuterol detection using SERS. Lorenzo *et al*. detected clenbuterol using SERS and investigated the absorption and aggregation of clenbuterol on Au and Ag nanoparticles at different pH values^20^. It was found that the adsorption of clenbuterol molecules was more effective on gold nanoparticles under acidic condition based on the direct interaction of the aromatic or aliphatic moieties through ionic or coordination bonds with the metal. But the sensitivity was low, with a limit of detection (LOD) of 35 ng·mL^−1^. Zhu *et al*. reported a SERS detection of clenbuterol based on a competitive SERS immunoassay with a LOD of 0.1 pg·mL^−1^ 15. The SERS probe was gold nanoparticle labeled by 4,4′- dipyridyl and clenbuterol antibody, and the detection was carried out by competitive binding between free clenbuterol and the antibody labeled SERS nanoprobe substrates. The method was ultrasensitive but the preparation of functional SERS probes was relatively complicated. Xie *et al*. prepared a modified Au^MBA^@Ag - Antibody probe (the notation “Au^MBA^@Ag” refers to the polyclonal antibody of clenbuterol labeled Au-Ag core-shell nanoparticles sandwiched with a Raman reporter 4-mercaptobenzoic acid (MBA)) and demonstrated a LOD of 0.24 pg·mL^−1^ for quantitative detection of clenbuterol from urine^14^. Thus, SERS can be used as a rapid, simple, and ultrasensitive analytical method for detecting clenbuterol in urine samples.

In this study, we designed a unique hybrid graphene oxide/Au nanoparticle(GO/AuNPs) SERS probe for clenbuterol detection from urine sample.The hybrid probe was stable within four weeks under 4 °C storage temperature, and the LOD and the limit of quantitation (LOQ) based on SERS for real urine sample were 0.5 ng·mL^−1^ and 1 ng·mL^−1^, respectively. A sufficient linearity (*R*^2^ = 0.976) was found in the concentration range of 1–20 ng·mL^−1^ in urine, and the recoveries of clenbuterol from spiked urine samples were in the range of 82.8–92.4% with relative standard deviation (RSD) values in range of 0.81–4.32% (*n*=6). The total detection time was approximately 15 min/sample.

## Results and Discussion

**Figure 1:**
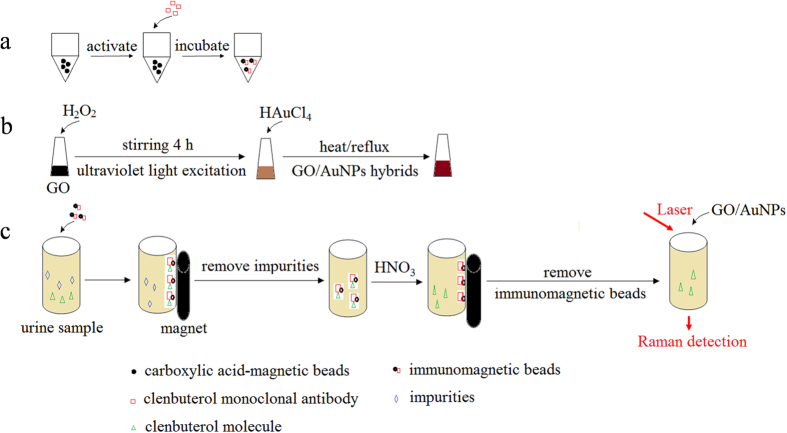
The schematics of clenbuterol detection: (**a**) Preparation of immunomagnetic beads; (**b**) Synthesis of GO/AuNPs hybrids; (**c**) Sample pretreatment and SERS measurement.

The general clenbuterol detection strategy can be divided into the following three steps as shown in Fig. 1: the preparation of immune-magnetic beads, the synthesis of GO/AuNPs hybrids, and a sample pretreatment and SERS measurement procedure. And some of the details are described in the “**Materials and Methods**” section. Below we discuss the most important results obtained.

### Characterization of GO/AuNPs hybrids

**Figure 2:**
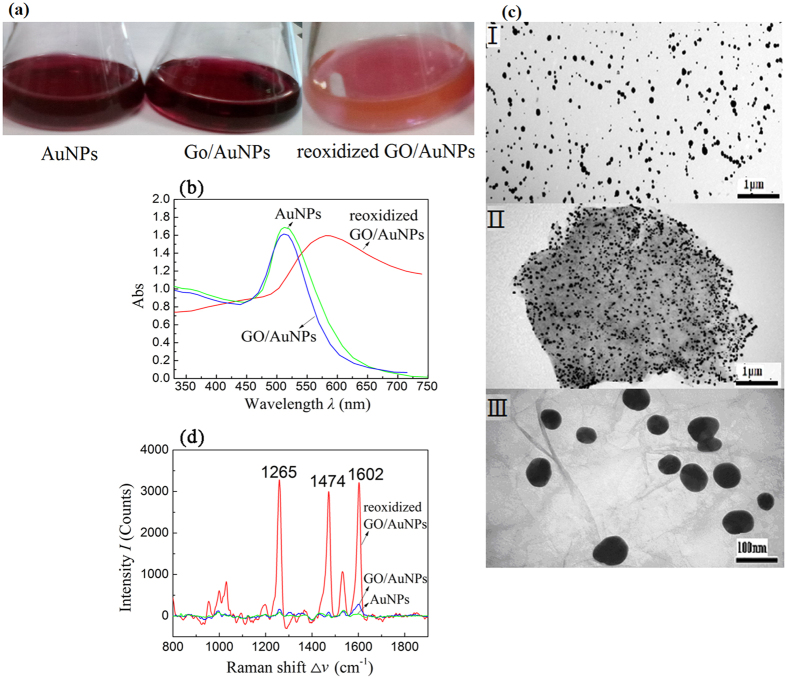
(**a**) Resuspended solutions of AuNPs, untreated GO/AuNPs and reoxizided graphene oxide (GO)/ gold nanoparticles (AuNPs) hybrids; (**b**)The UV-Vis spectra of gold nanoparticles (AuNPs), untreated graphene oxide (GO)/AuNPs hybrids, and reoxidized GO/AuNPs hybrids; (**c**) Transmission electron microscopy (TEM) images of (I) untreated GO/AuNPs hybrids, (II)reoxidized GO/AuNPs hybrids, and (III) AuNPs; (**d**) Characteristic SERS spectra of clenbuterol in the presence of (I) reoxidized graphene oxide (GO)/gold nanoparticles(AuNPs) hybrids, (II) untreated GO/AuNPs hybrids, and (III) AuNPs.

A photograph of the suspensions of AuNPs, untreated GO/AuNPs, and reoxizided GO/AuNPs is shown in Fig. 2(a). Both AuNPs and untreated GO/AuNPs have similar wine red color while the reoxidized GO/AuNPs shows a light red color, which means the absorbance peak wavelength of reoxidized GO/AuNPs has significantly red shifted, which is confirmed by the UV-Vis absorbance spectra shown in Fig. 2(b). Both AuNPs and untreated GO/AuNPs have a similar absorbance peak at *λ*_0_ = 530 nm, which is a typical localized surface plasmon resonance (LSPR) wavelength of AuNPs^21^. Since the untreated GO/AuNPs have the same LSPR wavelength as that of AuNPs, the AuNPs are either loosely attached on the GO sheets or not aggregate into closely packed AuNP clusters. The LSPR wavelength of the reoxidized GO/AuNPs red shifts to *λ*_0_ = 580 nm. Such a significant red shift could be due to the clustering of AuNPs or due to the plasmonic coupling between AuNPs and GO sheets^22^. However, the TEM images shown in Fig. 2(c) confirm that even there are AuNP clusters on GO sheets, the amount of AuNP clusters is rare. Therefore, such a red shift could be due to the plasmonic coupling between AuNPs and GO sheets - the reoxidization process made the AuNPs anchor more closely to the surface of GO sheets.

In fact reoxidized GO/AuNPs show the best SERS performance for detecting clenbuterol. 20 ng·mL^−1^ clenbuterol standard solution was used to evaluate the SERS performance of different solutions to detect clenbuterol, see Fig. 2(d). The most intense SERS peaks of the clenbuterol standard solution were at Δ*v* = 1265, 1474, and 1602 cm^−1^, respectively. The Δ*v* = 1474 cm^−1^ peak corresponds to the in-plane stretching vibrations coupled with the anilinic C-N stretching vibration; the Δ*v* = 1602 cm^−1^ peak is assigned to the C=C stretching band of the aromatic ring, and the Δ*v* = 1265 cm^−1^ peak results from the anilinic C-N stretching coupled with in-plane ring deformations^20^. The SERS peak intensity *I*_1474_ at Δ*v* = 1474 cm^−1^ is 2986 counts for the reoxidized GO/AuNPs, as compared to 88 counts for the GO/AuNPs. Thus, the second oxidization treatment is indeed very necessary to improve the SERS detection sensitivity. Therefore, we choose the reoxidized GO/AuNPs for the clenbuterol detection.

### SERS detection of clenbuterol

**Figure 3:**
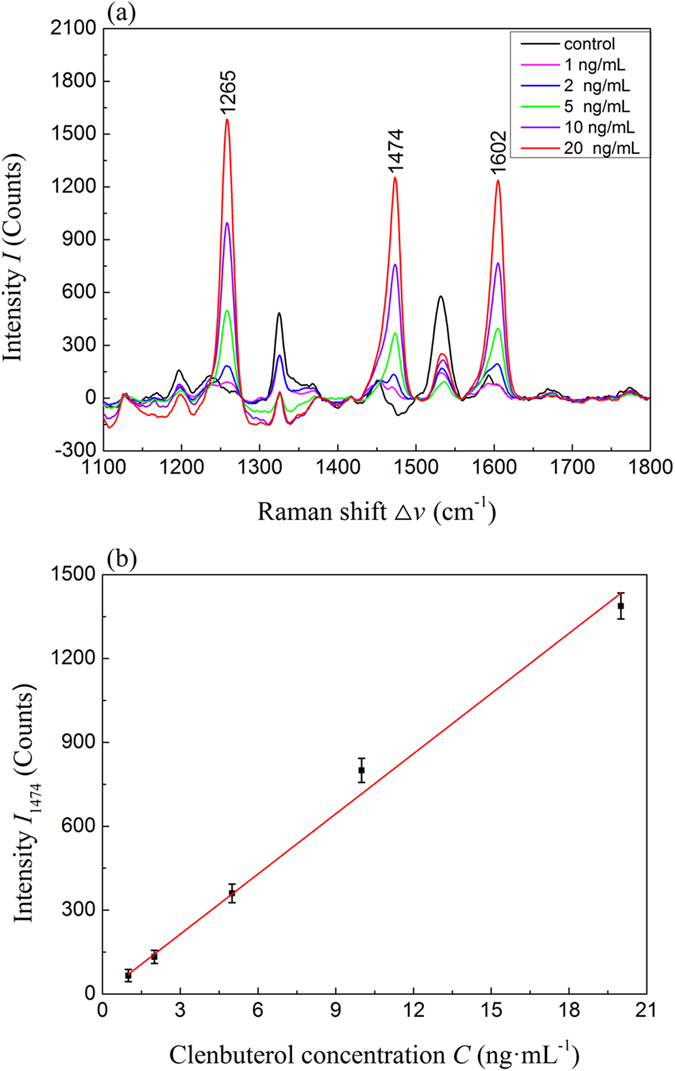
(**a**) SERS spectra of clenbuterol at different standard solution concentrations (*C* = 1, 2, 5, 10, and 20 ng•mL^−1^. Each spectrum is the average of 6 different measurement; (**b**) The plot of *I*_1474_ versus *C*.

The LOD of SERS on clenbuterol detection has been evaluated through the SERS measurements of different concentrations of clenbuterol standard solutions *C* = 1, 2, 5, 10, and 20 ng·mL^−1^ (*n* = 6) and clenbuterol inoculated urine samples. Figure 3(a) shows the average spectra of clenbuterol standard solutions. As one can see, the control sample shows significant peaks at Δ*v* = 1236, 1325, 1532, and 1593 cm^−1^. Those peaks may correspond to the vibrations of the solvent molecules or the background in the control samples. However, for all the spectra of clenbuterol, the three characteristic peaks at Δ*v* = 1265, 1474, and 1602 cm^−1^ appear, and the intensities of these peaks increase as the *C* increases. To quantify the SERS peak intensity and clenbuterol concentration, we plot the peak intensity at Δ*v* = 1474 cm^−1^, *I*_1474_, as a function of *C* in Fig. 3(b). Clearly *I*_1474_ versus *C* follows a linear relationship, and a liner fit shown as the solid line in Fig. 3(b) gives a slope of 71.7 counts·mL/ng, which corresponding to the sensitivity of SERS detection. The sensitivity for the standard solution is 0.014 ng·mL^−1^/intensity count. The limit of detection (LOD) is the lowest concentration at which *I*_1474_ is significantly larger than that from a control sample. We use 3-sigma rule of the SERS spectra from a control sample to set the cut-off *I*_1474_: the average SERS intensity value at Δ*v* = 1474 cm^−1^ plus three times the standard deviation of SERS intensity of the control sample^23^. Similarly, the limit of quantification (LOQ) is determined from the average SERS intensity at Δ*v* = 1474 cm^−1^ plus ten times the standard deviation of SERS intensity of a control sample. From our measurements for the purified clenbuterol solution, LOD is approximately 0.5 ng·mL^−1^ and the LOQ is1 ng·mL^−1^.

**Figure 4:**
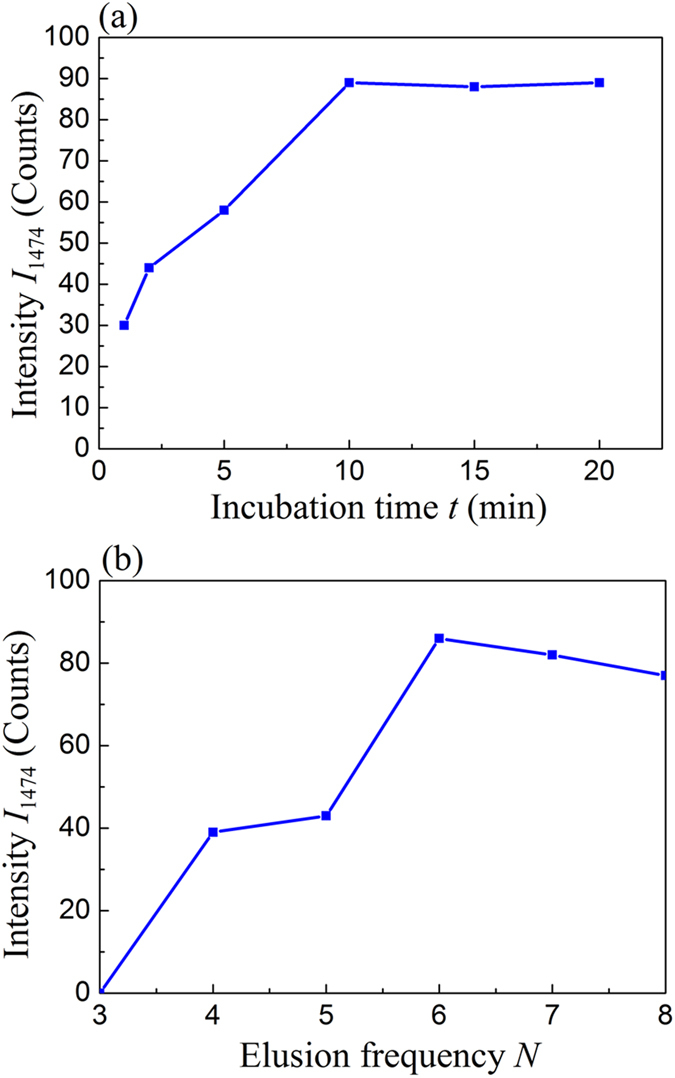
(**a**) The SERS intensity *I*_1474_ versus incubation time t; (**b**) The SERS intensity *I*_1474_ versus Ab-MBs elusion frequencies *N*.

The LOD from urine sample is determined by three primary factors, the clenbuterol extraction efficiency from urine, the potential interference from contaminants in the extracted sample, and the intrinsic SERS detection LOD of pure clenbuterol. Urine samples are complex matrices containing multiple contents, such as urea, creatinine, uric acid, and small proteins. Many of them could generate SERS signals if not removed, and will introduce interference for clenbuterol detection. In order to remove the interference in the urine and obtain clenbuterol SERS spectra with a high signal-to-noise ratio, we use the Ab-MBs to extract the clenbuterol molecules from urine. For such an extraction process, the incubation time *t* and the elution frequency play the most important roles. The incubation is required to allow the clenbuterol antibodies on the magnetic beads to have enough time to capture the clenbuterol molecules in urine as much as possible. To investigate the effect of the incubation time on SERS detection of clenbuterol from urine, the urine sample inoculated with 1 ng·mL^−1^ of clenbuterol was used with a fixed washing time *N* = 6 and the Ab-MBs incubation time in urine samples was varied as *t* = 1, 2, 5, 10, 15, and 20 min, respectively. The SERS intensity (*I*_1474_) of the extracted clenbuterol as a function of *t* is plotted in Fig. 4(a). Initially *I*_1474_ increases almost linearly with *t*. When *t* = 10 min, *I*_1474_ reaches a maximum. Further increase of *t* does not increase the SERS intensity. Therefore, *t* = 10 min is the optimum incubation time. The elution is required to further remove contaminants in the Ab-MBs extracted from urine. Such a process not only can reduce the contaminants, but also could remove captured clenbuterol on Ab-MBs. Therefore, there must be an optimal elusion frequency to retain most of the clenbuterol while to remove most of the contaminants. By keeping the same inoculated urine sample and fixing *t* = 10 min, the relationship between the SERS intensity *I*_1474_ and the elusion frequency *N* (the Ab-MBs were eluded with *N* = 3, 4, 5, 6, 7, or 8 aliquots of 1.5 mL of DI water) was investigated, and is plotted in Fig. 4(b). Clearly *N*= 6 is the optimal elution frequency.

**Figure 5:**
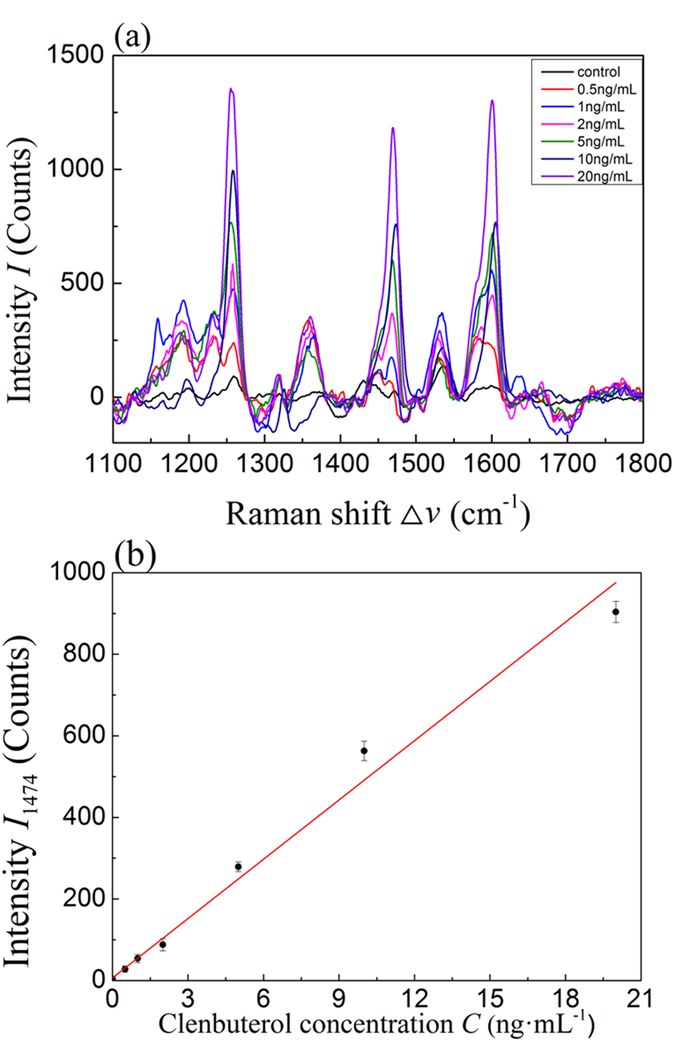
(**a**) SERS spectra of the extracted clenbuterol inoculated urine samples; (**b**) The plot of *I*_1474_ versus the inoculated clenbuterol concentration *C*.

Under the optimal sample treatment condition, *t* =10 min and *N* = 6, the quantitative relationship between the SERS spectra of the extracted clenbuterol and the inoculated clenbuterol concentration *C* = 0.5, 1, 2, 5, 10, and 20 ng·mL^−1^, respectively, in urine was investigated. Figure 5(a) shows the clenbuterol concentration dependent SERS spectra, and they show all the characteristic peaks of clenbuterol SERS as compared to Fig. 2(a). However, compared to Fig. 2(a), the spectra in Fig. 5(a) have more background noise. When plot the *I*_1474_ as a function of *C*, as shown in Fig. 5(b), a linear relationship is also obtained, like Fig. 3(b). Such a linear relationship demonstrates that the proposed SERS method can still quantitatively determine the amount of clenbuterol in urine. A linear fitting reveals that the slope is 47.4 counts·mL/ng, which is about 2/3th of the value (71.7 counts·mL/ng) obtained from Fig. 3(b). Such a difference indicates that background matrix in urine samples is affecting the detection. To eliminate the effect of the matrix in the proposed method, matrix-matched calibration standard curves were selected to quantify clenbuterol in the urine samples. Therefore, for practical application of the SERS detection method, we will use Fig. 5(b) as the calibration curve to account for the effect of background in urine. The sensitivity for the urine samples is 0.021 ng·mL^−1^/intensity count, which is comparable to that of the standard solution. Based on the previously stated criteria, we determine that the LOD is approximately 0.5 ng·mL^−1^ and the LOQ is 1 ng·mL^−1^ for the inoculated urine samples.

### Validation of the proposed method with real urine samples

**Figure 6:**
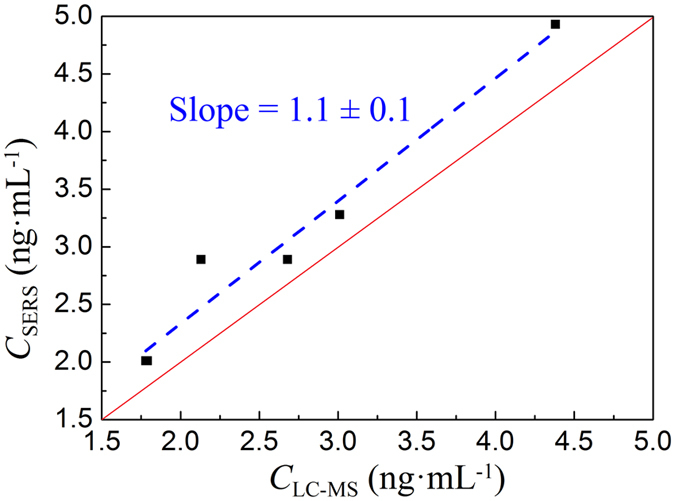
The plot of the clenbuterol concentration *C*_SERS_ determined by SERS versus the clenbuterol concentration *C*_LC-MS_ determined by LC-MS/MS. The solid line represent *C*_SERS_ = *C*_LC-MS_.

The practical application of the proposed SERS method for clenbuterol detection is validated using the real urine samples. 40 different urine samples from sheep, pig, and cow obtained from the Chinese National Feed Supervision from 2012–2014, previously tested by LC-MS/MS^9^, were used for SERS measurements. We adapted the optimal Ab-MBs treatment methods, identified the SERS peak intensity *I*_1474_, applied the LOD criteria, and used Fig. 5(b) as the calibration curve. The results are summarized in Table 1. Comparing to LC-MS/MS method, SERS can identify all the positive and negative urine samples with 100% accuracy. For all the positive samples, the SERS predicted clenbuterol concentration *C*_SERS_ in urine is very close to those determined by LC-MS/MS, *C*_LC-MS_. Figure 6 plots *C*_SERS_ versus *C*_LC-MS_. Clearly the data follows a linear relationship with a slope of ~1.1. In all the cases, SERS gave a slightly higher concentration than that determined by LC-MS/MS. However, a slope of 1.1 demonstrates that SERS results are highly correlated to LC-MS/MS results.

### Addressing practical concerns of the SERS assay

To further validate the SERS detection method, clenbuterol recoveries experiment were carried. The urine samples spiked with 1, 2, 5, 10, and 20 ng·mL^−1^ of clenbuterol were prepared. We adapted the optimal Ab-MBs treatment methods to remove the interferences of the urine samples, and did the SERS measurement as mentioned above. The analysis results was expressed as “detected values” (ng·mL^−1^). The recoveries values were the ratio between the detected values and the spiked values. The recoveries were found to be 92.4, 83.4, 91.5, 88.4, and 82.8%, respectively (Table 2). These results indicate that the SERS based clenbuteral method is reliable and is fully compatible with the standards recommended by EU^24^.

Two other critical issues for SERS to be used as a practical sensor are the reproducibility of SERS substrates and their shelf time. In our study, the protocols for the preparation of the GO/AuNPs hybrids substrate were well controlled. For example, the GO/AuNPs hybrids substrate storage conditions (Fig. S1, Supporting Information), the vortexing duration of the GO/AuNPs hybrids before use (Fig. S2, Supporting Information), the vortexing time for sample mixing (Fig. S3, Supporting Information), and the cleaning of the glass bottles used for the SERS detection (Fig. S4, Supporting Information), are the main factors influencing the aggregation of the GO/AuNPs hybrids and affecting the reproducibility of the SERS measurements, and we have set a strict protocol based on all these results. Once the protocol has been followed, the intensity of the clenbuterol characteristic peak at Δ*v* = 1474 cm^−1^ is very stable when GO/AuNPs hybrids was stored under 4 °C for at least 4 weeks. In addition, the inter- and intra- day detection precisions of the SERS detection method were determined for urine samples spiked with 2 ng·mL^−1^ of clenbuterol, and the results are summarized in Table 3.The relative standard deviation (RSD) was used to indicate the reproducibility of the detection results, and were found to be in between 2.89% and 6.41%. These low RSD values demonstrate that the proposed SERS method is highly reproducible and reliable.

### Specificity of clenbuterol detection

The specificity of the present method was explored against two other kinds of β_2_-adrenergic agonists: ractopamine and salbutamol, which are the common “lean meat powder” that could coexist in animal urine samples. Fig. S5 of Supporting Information shows the standard SERS spectra of clenbuterol, ractopamine and salbutamol with the concentrations of 10 ng·mL^−1^. The SERS spectrum of ractopamine has two characteristic peaks at Δ*v* = 650 and 838 cm^−1^ while salbutamol shows two characteristic peaks at Δ*v* = 950 and 1048 cm^−1^, respectively. These characteristic peaks are similar to those reported in the literatures^20^,^25^,^26^, but are very different from the characteristic peaks of the clenbuterol (Δ*v* = 1265, 1474, and 1602 cm^−1^), as summarized in Table S1 of the Supporting Information. Thus, one could use the presence of these characteristic peaks to assess the specificity of the proposed detection method. Five mixtures of clenbuterol, ractopamine, and salbutamol in real urine samples with the equal concentrations of 1, 2, 5, 10, and 20 ng·mL^−1^ for each individual agonist respectively, were used in the specificity test. After the Ab-MBs treatment, the SERS spectra were shown in Fig. S6(a) of Supporting Information. Only the three clenbuterol peaks dominate the spectra and no characteristic peaks of ractopamine and salbutamol are presented, due to the high specific binding efficiency of Ab-MBs treatment. In addition, Fig. S6(b) shows the SERS intensity of the Δ*v* = 1474 cm^−1^ peak versus the clenbuterol concentration, and the sensitivity is 0.020 ng·mL^−1^/intensity count, which is almost the same as those shown in Fig. 5(b). Therefore, the qualitative and quantitative analysis of clenbuterol in urine samples were not affected by ractopamine and salbutamol. All the results indicated that this method has a high specificity for the detection of clenbuterol in real urine samples.

## Materials and Methods

### Materials and reagents

The non-contaminated urine samples used in the study were obtained from Small Tailed Han Sheep and were tested by LC-MS/MS. Clenbuterol hydrochloride and clenbuterol monoclonal antibody were purchased from Dr. Ehrenstorfer GmbH and Kwinbon Biotechnology, respectively. Chloroauric acid tetrahydrate (HAuCl_4_·4H_2_O, analytical grade, >99.9%) and sodium citrate (analytical grade) were acquired from the Sinopharm Chemical Reagent Co., Ltd.Graphene Oxide (GO) solution (1 g·L^−1^, XFNANO Co., Ltd.), carboxylic acid-magnetic beads (2.8 μm diameter, 30 mg·mL^−1^, Life Technologies, Thermo Fisher Scientific Inc.), analytical grade bovine serum albumin (BSA), 2-(N-Morpholino) ethanesulfonic acid (MES), 1-ethyl-3-(3-dimethylaminopropyl) carbodiimide hydrochloride (EDC), N-hydroxysuccinimide (NHS), and all other chemicals from Sigma Aldrich Co., Ltd. were used as received. Ultra-pure water (Milli-Q, Millipore Corp.) was used in all experiments. All of the glass vials were cleaned using a concentrated piranha solution (95% sulfuric acid/30% hydrogen peroxide solution, v/v = 7:3), then rinsed twice using ultra-pure water, and dried for 2 h under airflow in an oven at 100 °C.

### Preparation of immunomagnetic beads

To activate the immunomagnetic beads, 100 μL carboxylic acid-magnetic beads thoroughly resuspended were washed by adding 100 μL of a 25 mM MES buffer solution at pH 5 and incubated for 10 min, and a 50 μL aliquot of 50 mg·mL^−1^NHS solution and a 50 μL aliquot of the well-mixed suspension of 50 mg·mL^−1^ EDC solution (in a cold buffer solution) were added. The solution was incubated with vortex mixer at ambient temperature for 30 min. After incubation, magnetic beads were separated from the solution, and were washed twice. Then, a 10 μL aliquot of the clenbuterol monoclonal antibody was added to the magnetic beads, and 25 mM MES buffer (pH = 5) was added, to a final volume of 100 μL. The solution was incubated for no less than 30 min at ambient temperature with a vortex mixer. Finally, the antibody functionalized magnetic beads (Ab-MBs) were collected from the solution.

To block any unreacted carboxylic acid groups, the Ab-MBs were incubated in a 100 μL 50 mM Tris buffer solution at pH = 7.4 for 15 min and washed four times in Tris buffer. The resulting beads were resuspended in 200 μL phosphate buffer solution (PBS) solution and were stored in the dark at 2–8 °C for three months.

### Synthesis of GO/AuNPs hybrids

20 mL 0.2 g·L^−1^ GO solution was added to 20 mL 30% H_2_O_2_ solution at room temperature, followed by stirring for 4 h under ultraviolet light excitation (Portable ultraviolet radiation light, WFH-204B, 254 nm, 50 HZ). The resulting 3.5 mL GO solution were mixed with 350 μL 1 wt % aqueous sodium citrate and 40 mL distilled water in a 100 mL Erlenmeyer flask. With 800 rpm vigorous stirring the solution was heated to reflux, and 500 μL 1 wt % HAuCl_4_ was added within 2 s. The solution remained under reflux for another 10 min, then the heating source was removed while the solution was kept continuously stirring and cooled down to ambient temperature within 10 min. The resultant GO/AuNPs hybrids were washed twice by distilled water after centrifugation at 2800 rpm for 5 min (DAIHAN WiseSpin CF-10), and were stored at 4 °C for no longer than four weeks (the storage time on SERS response is shown in Fig. S1, Supporting Information). The GO/AuNPs were characterized by a transmission electron microscopy (TEM) (Hitachi H-7500) and a UV-Vis spectrometer (Shimadzu UV-2700). Before use, the GO/AuNPs were gently vortexed for 1 min (the effect of vortexing time on SERS response is shown in Fig. S2, Supporting Information).

### Sample pretreatment

A 50 μL Ab-MB stock solution was washed twice with 1.5 mL deionized (DI) water, then mixed with a 2 mL urine sample. After shaking the tube for 60 s, the mixture was incubated at ambient temperature for 10 min and washed six times with 1.5 mL DI water. Finally, 1 mL HNO_3_ (1 wt %) was added, and the Ab-MBs were extracted by a permanent magnet. The remained solution was used for SERS measurements and detection.

### SERS measurement

60 μL of above solution was gently mixed with 600 μL GO/AuNPs for 10 s (Fig. S3, Supporting Information), then the suspension was analyzed by a portable Raman spectrophotometer (Raman Tracer-200-HS) at 200 mW power and 10 s exposure time with an incident laser wavelength of 785 nm and a spectral resolution of 4 cm^−1^. Each spectrum was an average of two scans.

### Data analysis

The SERS spectra ranging from 500–2500 cm^−1^ were analyzed using the RamanAnalyzer software (OptoTrace Technologies, Inc.). The spectra baseline was offset using the Savitzky-Golay second derivative transformation^27^. Other pre-processing algorithms, such as polynomial subtraction and smoothening, were also used.

### Ethics Statement

The study was approved by the Animal Research Ethics Committee of Chinese Ministry of Agriculture and supported by a special fund for Chinese Agro-scientific Research in the Public Interest (No. 201203088-1). The whole experiment was carried out in accordance with the guidelines of the animal experiment and some relevant bulletins published by the ministry of agriculture of the People’s Republic of China. The urine samples were obtained from the Chinese National Feed Supervision Mission from 2012–2014.

## Conclusions

SERS is an emerging analytical technique with satisfactory performance in the detection of trace amount of targeted analyte. Herein, based on a unique GO/AuNPs hybrids, a rapid, selective, and sensitive SERS method combined with immunomagnetic was developed to detect the clenbuterol content in animal urine. We demonstrated that based on a linear calibration curve of the SERS characteristic peak intensity of clenbuterol at Δ*v* = 1474 cm^−1^ *versus* the spiked clenbuterol concentration, the quantity of clenbuterol in real animal urine samples can be determined. These results matched well with those determined by LC-MS/MS. The limits of detection and quantification for detecting clenbuterol from the urine samples were 0.5 ng·mL^−1^ and 1 ng·mL^−1^, respectively, and the recovery clenbuterol rates were 82.8–92.4% with coefficients of variation <9.4%. Furthermore, the day-to-day variation of the detection was less than 6.41%, and the shelving life of the SERS substrates is no less than 4 weeks.

Currently, the Chinese government surveys the illicit use of clenbuterol in animal farming using the GC-MS or LC-MS/MS methods. These standard methods not only require time-consuming sample pretreatment, with approximately 30 min/sample, but also need expensive chromatography instruments operated by experienced, well-trained personal. The SERS method we developed here only require a portable Raman instrument and the specific designed Ab-MBs & GO/AuNP assays with similar sensitivity, while the total detection time is only approximately 15 min/sample. Thus, the proposed SERS method holds a great promise for field-based clenbuterol detection in animal farming.This protocol could be developed for routine on-site monitoring of urine for the purposes of government and market surveillance.

## Additional Information

**How to cite this article**: Cheng, J. *et al*. Highly Sensitive Detection of Clenbuterol in Animal Urine Using Immunomagnetic Bead Treatment and Surface-Enhanced Raman Spectroscopy. *Sci. Rep.* **6**, 32637; doi: 10.1038/srep32637 (2016).

## Supplementary Material

Supplementary Information

## Figures and Tables

**Table 1:** Comparison between LC-MS/MS and SERS results for forty animal urine samples.

Sample Number	LC-MS/MS (ng·mL^−1^)	SERS	
		Quantity (ng·mL^−1^)^a^	Quality^b^
ZN20120234	ND	ND	Negative
ZN20120368	ND	ND	Negative
ZN20120190	ND	ND	Negative
NN20120191	ND	ND	Negative
NN20122167	ND	ND	Negative
YN20122346	2.13	2.89	Positive
YN20122439	ND	ND	Negative
ZN20122591	ND	ND	Negative
NN20122680	ND	ND	Negative
YN20122681	ND	ND	Negative
NN20130004	ND	ND	Negative
ZN20130007	1.78	2.01	Positive
ZN20130008	4.38	4.93	Positive
YN20130130	ND	ND	Negative
YN20130133	ND	ND	Negative
YN20130134	ND	ND	Negative
YN20130154	ND	ND	Negative
NN20130168	ND	ND	Negative
NN20130169	ND	ND	Negative
ZN20130170	ND	ND	Negative
NN20130201	3.01	3.28	Positive
YN20130217	ND	ND	Negative
YN20130239	ND	ND	Negative
NN20130298	ND	ND	Negative
NN20130303	ND	ND	Negative
ZN20130312	ND	ND	Negative
NN20130341	ND	ND	Negative
NY20130342	ND	ND	Negative
ZN20130343	ND	ND	Negative
ZN20130344	ND	ND	Negative
NN20130345	1.79	2.01	Positive
NN20130346	ND	ND	Negative
ZN20140001	ND	ND	Negative
NN20140010	ND	ND	Negative
NN20140012	ND	ND	Negative
ZN20140034	ND	ND	Negative
YN20140035	2.68	2.89	Positive
YN20140055	ND	ND	Negative
YN20140056	ND	ND	Negative
NN20140193	ND	ND	Negative

^a^ND means the clenbuterol content was below the limit of detection of the method (0.5 ng·mL^−1^).

^b^A negative result means the content of clenbuterol in the sample was <1.0 ng·mL^−1^.

**Table 2:** Recovery of clenbuterol from spiked urine samples.

Spiked clenbuterolcon-centration (ng·mL^−1^)	Average SERS peak intensity*I*_1474_(counts)	Amount detected^a^ (ng·mL^−1^)	Recovery (%) (n = 6)	RSD^b^ (%)
1	54.31	0.92 ± 0.04	92.4	4.32
2	88.39	1.66 ± 0.02	83.4	1.28
5	279.01	4.58 ± 0.08	91.5	1.82
10	563.48	8.84 ± 0.07	88.4	0.81
20	904.22	16.56 ± 0.23	82.8	1.39

^a^Average values from six determinations for each concentration.

^b^Relative standard deviation of peak intensity (RSD (%) = (SD/mean) × 100).

**Table 3:** Inter- and intra-day reproducibility of the detection using urine sampled spiked with a 2 ng·mL^−1^ clenbuterol.

Analysis^a^	Measurements	Amount detected^b^ (ng·mL^−1^)	Average SERS peak intensity*I*_1474_^a^ (counts)	RSD^b^ (%)	Overall RSD^c^ (%)
Intra-day	Day 1	1.89 ± 0.09	88.31	4.7	2.89
	Day 1	1.95 ± 0.08	92.38	4.0	
	Day 1	1.97 ± 0.12	93.24	6.0	
	Day 1	2.02 ± 0.20	94.09	9.4	
	Day 1	1.94 ± 0.10	91.27	4.7	
Inter-day	Day 1	1.92 ± 0.10	90.10	5.2	6.41
	Day 2	1.88 ± 0.09	88.01	4.6	
	Day 3	1.90 ± 0.07	89.90	3.6	
	Day 4	1.81 ± 0.07	78.04	3.7	
	Day 5	2.09 ± 0.15	95.20	7.1	

^a^Average of three determinations for each measurement.

^b^Relative standard deviation (RSD (%) = (SD/mean) × 100) of individual measurements or days.

^c^RSD % of inter- and intraday measurements.
