# Supplementary material for: Highly Sensitive Detection of Clenbuterol in Animal Urine Using Immunomagnetic Bead Treatment and Surface-Enhanced Raman Spectroscopy
Source: Sci Rep. 2016 Sep 7;6:32637. doi: 10.1038/srep32637 (PMC5013518; doi:10.1038/srep32637)
Supplement: Supplementary Information [file srep32637-s1.doc]

**Highly Sensitive Detection of Clenbuterol in Animal Urine Using Immunomagnetic Bead Treatment and Surface-Enhanced Raman Spectroscopy**

Jie Cheng^1^, Xiao-Ou Su^1^*, Shi Wang^1^, and Yiping Zhao^2^*

^1^ Institute of Quality Standards and Testing Technologies for Agro-products, Chinese Academy of Agricultural Sciences, Beijing, 100081, China

^2^ Department of Physics and Astronomy, University of Georgia, Athens, Georgia 30602, USA

*Corresponding author

E-mail: suxiaoou@caas.cn；zhaoy@physast.uga.edu

**Supporting Information**

**S1. GO/AuNPs hybrids storage conditions**

For the storage-temperature stability study, aliquots of freshly prepared GO/AuNPs hybrids (25 mL) were stored in 50 mL polypropylene centrifugation tubes under four different storage conditions: 0, 4, 20, and 30°C for 1, 2, 3, 4, 5, and 6 wk. Each week, SERS measurements of the clenbuterol standard solution (1 ng·mL^−1^) on the stored colloids were performed. The same measurement protocol for SERS analysis was used as detailed in the main text. Six repeat measurements per storage condition were acquired each week.

Figure S1 shows the effect of different storage conditions on the SERS intensity of the clenbuterol *I*_1474_. From the figure, we determined that the optimal storage temperature was 4°C, at which *I*_1474_ remained relatively stable (the intensity changed from 60.49 to 60.08 over 4 weeks). Storage temperatures above or below 4°C had severe effects on *I*_1474_. As the storage time increased, *I*_1474_ decreased, which may reflect the self-aggregation of the GO/AuNPs hybirds. These results are similar to those previously reported in the literature [1]. The acceptable storage time was less than 4 wk.


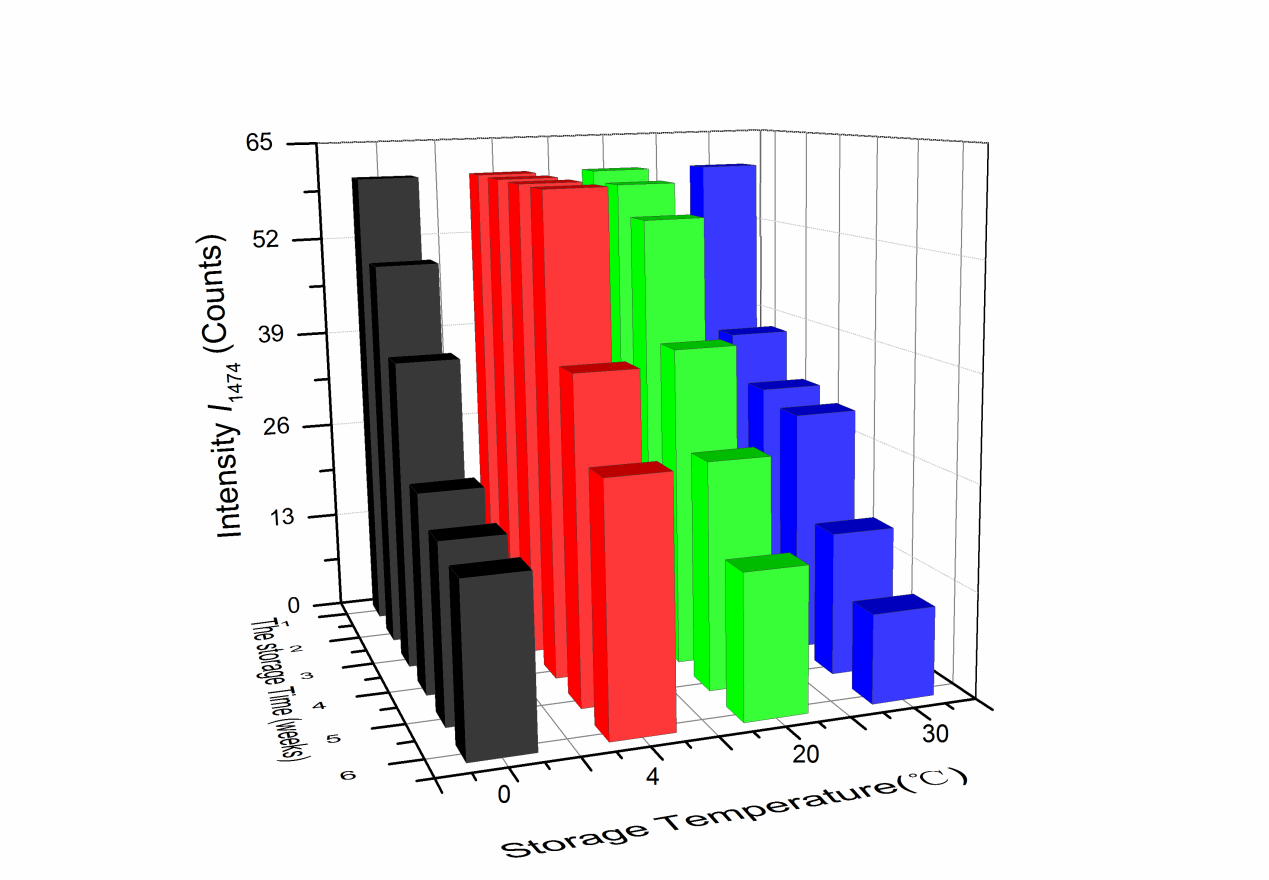


**Fig. S1.** The different storage conditions versus *I*_1474_

**S2. Effects of vortexing duration**

To perform the SERS analysis, the prepared GO/AuNPs hybrids solution (600 μL, gently vortexed at 1250 rpm for 1 min) and the clenbuterol standard solution (60 μL, 20 ng·mL^−1^) were transferred into the glass vial. The whole mixture was vortexed (1250 rpm) for 10 s before SERS detection. Variations of the above procedures were performed to study the effect of the vortexing times of the GO/AuNPs hybrids on clenbuterol SERS measurement. We varied the durations of vortexing (1) of the GO/AuNPs hybrids (0.5, 1, 1.5, 2, and 2.5 min); and (2) of the mixture of the GO/AuNPs hybrids and clenbuterol before SERS detection (2, 5, 10, 15, and 20 s).

Figure S2 shows the different vortexing times (at 1250 rpm for 0.5, 1, 1.5, 2, and 2.5 min) of the GO/AuNPs hybirds (after less than 4 wk storage at 4°C) versus *I*_1474_. Four replicates were measured. As shown in Fig. S2, an optimal vortexing time of 1 min was selected. Figure S3 shows the effects of vortexing duration for the sample mixtures (at 1250 rpm for 2, 5, 10, 15, and 20 s) on *I*_1474_. In this case, four replications were performed for each duration. The optimal mixing time was 10 s.


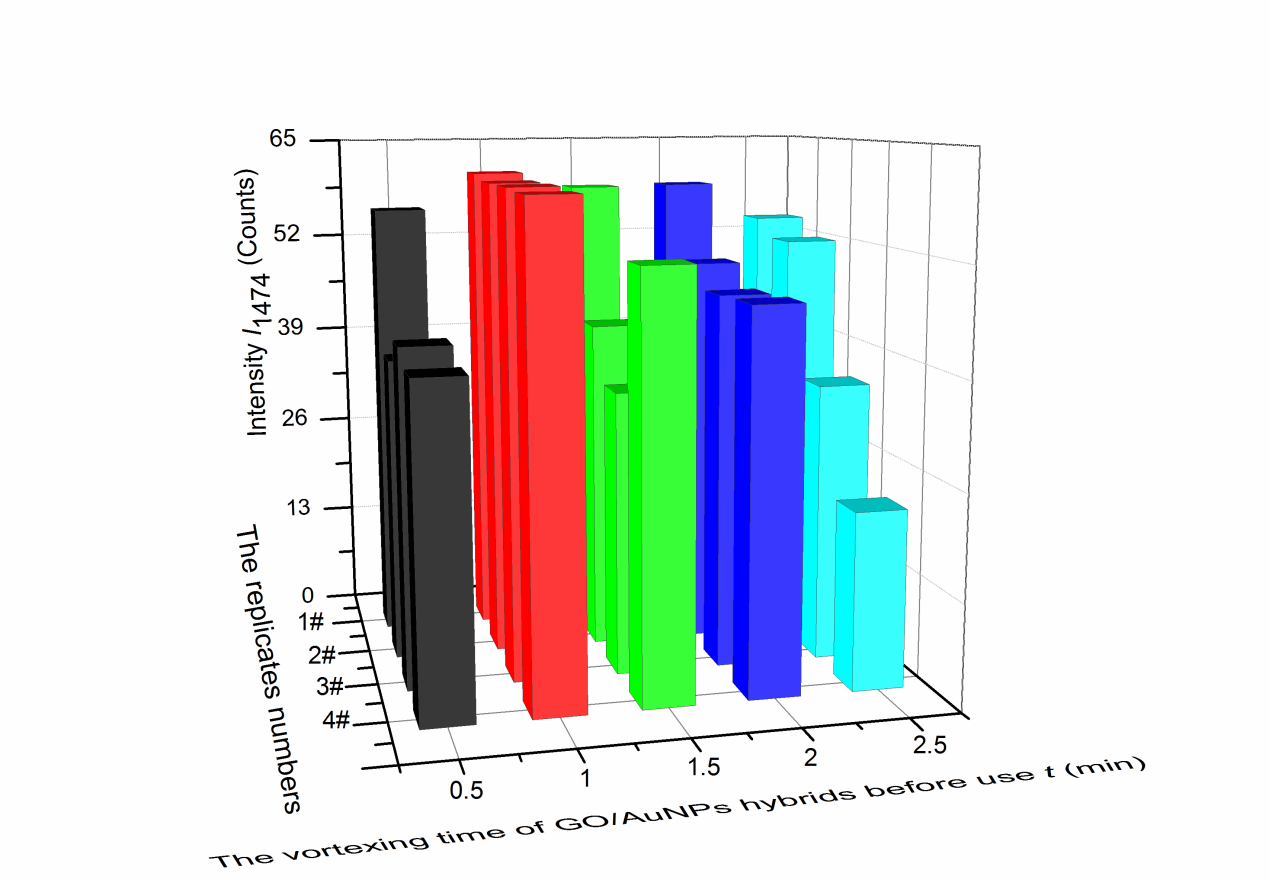


**Fig. S2.** The different pre-use vortexing times (at 1250 rpm for 0.5, 1, 1.5, 2, and 2.5 min) of the GO/AuNPs hybrids versus *I*_1474_

**
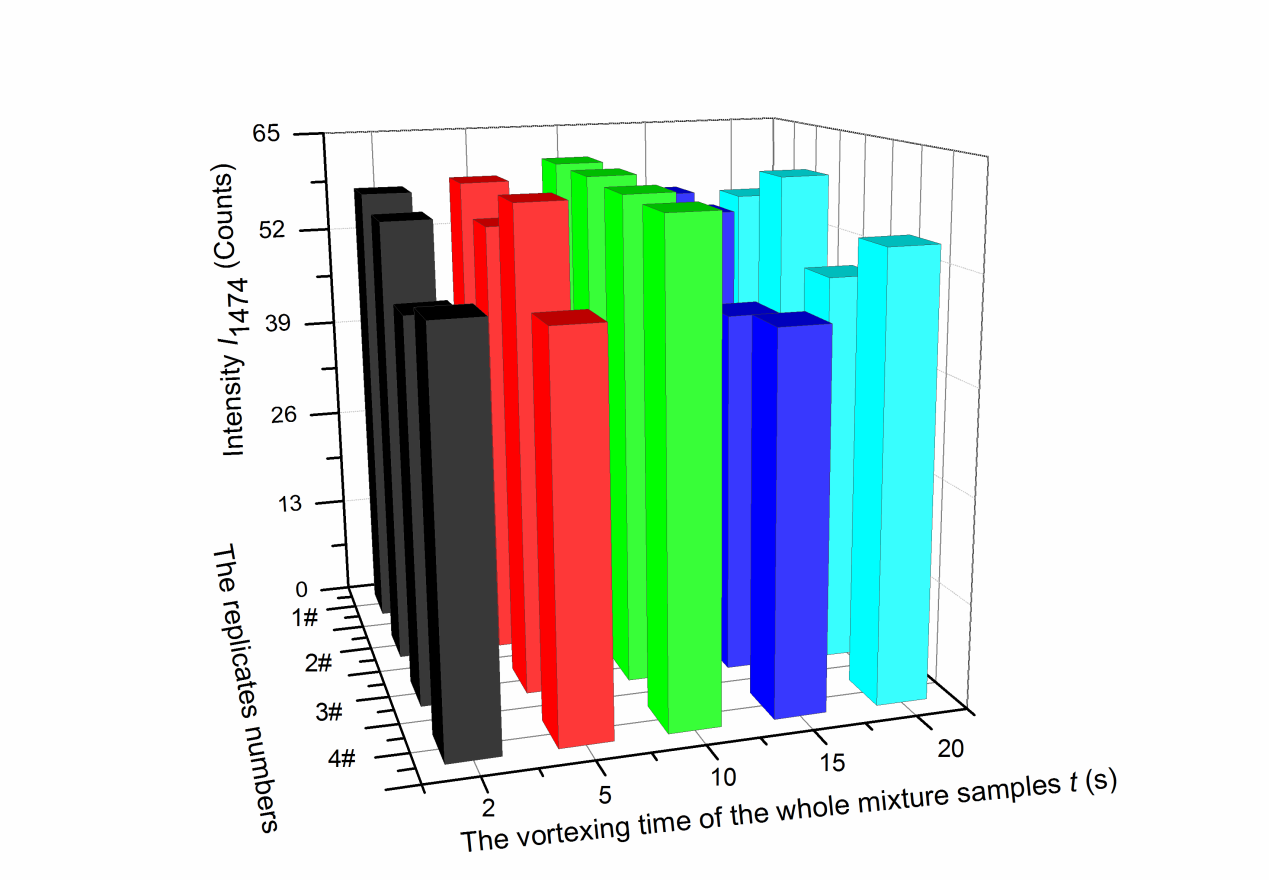
**

**Fig. S3.** The effect of different vortexing times of the sample mixtures (at 1250 rpm for 2, 5, 10, 15, and 20 s) on *I*_1474_

**S3. Glass vial cleaning protocol**

After the tests, the glass vials must be washed and dried for the next use. The selection of the cleaning solvent was quite important, as it affected the reproducibility of SERS measurements. We washed glass vials 4 times. After each wash time, SERS measurements of the clenbuterol standard solution (1 ng·mL^−1^) on the same washed glass vials were performed. Common cleaning solvents such as deionized water, methanol, aqua regia, and potassium bichromate were not very effective. The intensity of the characteristic signal at 1474 cm^−1^ decreased rapidly after each cleaning (Fig. S4).

The concentrated piranha solution (95% sulfuric acid/30% hydrogen peroxide solution (v/v = 7:3)) [2] was selected as the cleaning solvent. Because of the strongly oxidizing properties of the cleaning solution, the AuNPs and other contaminants were oxidized and effectively removed from the glass vials.


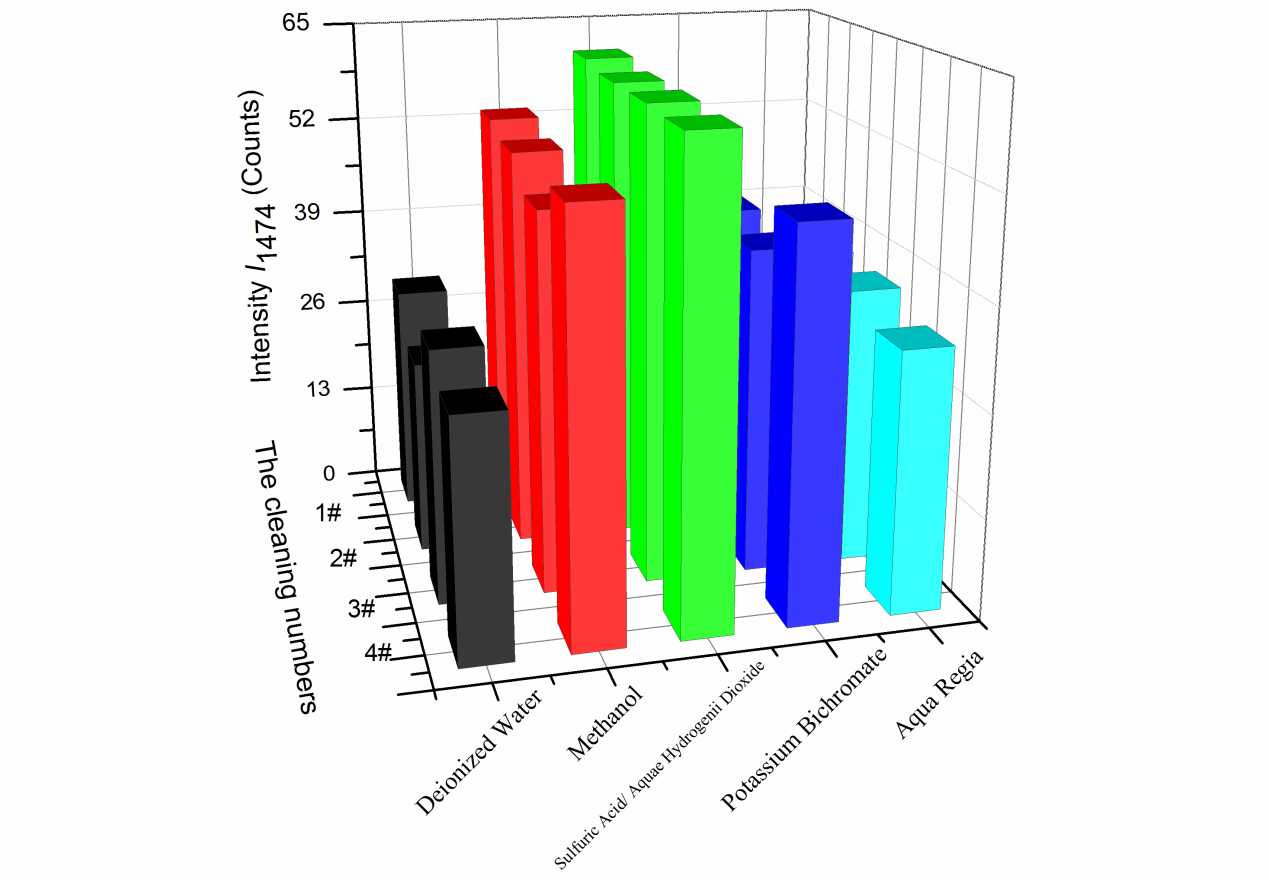


**Fig. S4.** Effects of vial cleaning solvent. *I*_1474_ decreased rapidly after each cleaning

**S4. Specificity test results**

**
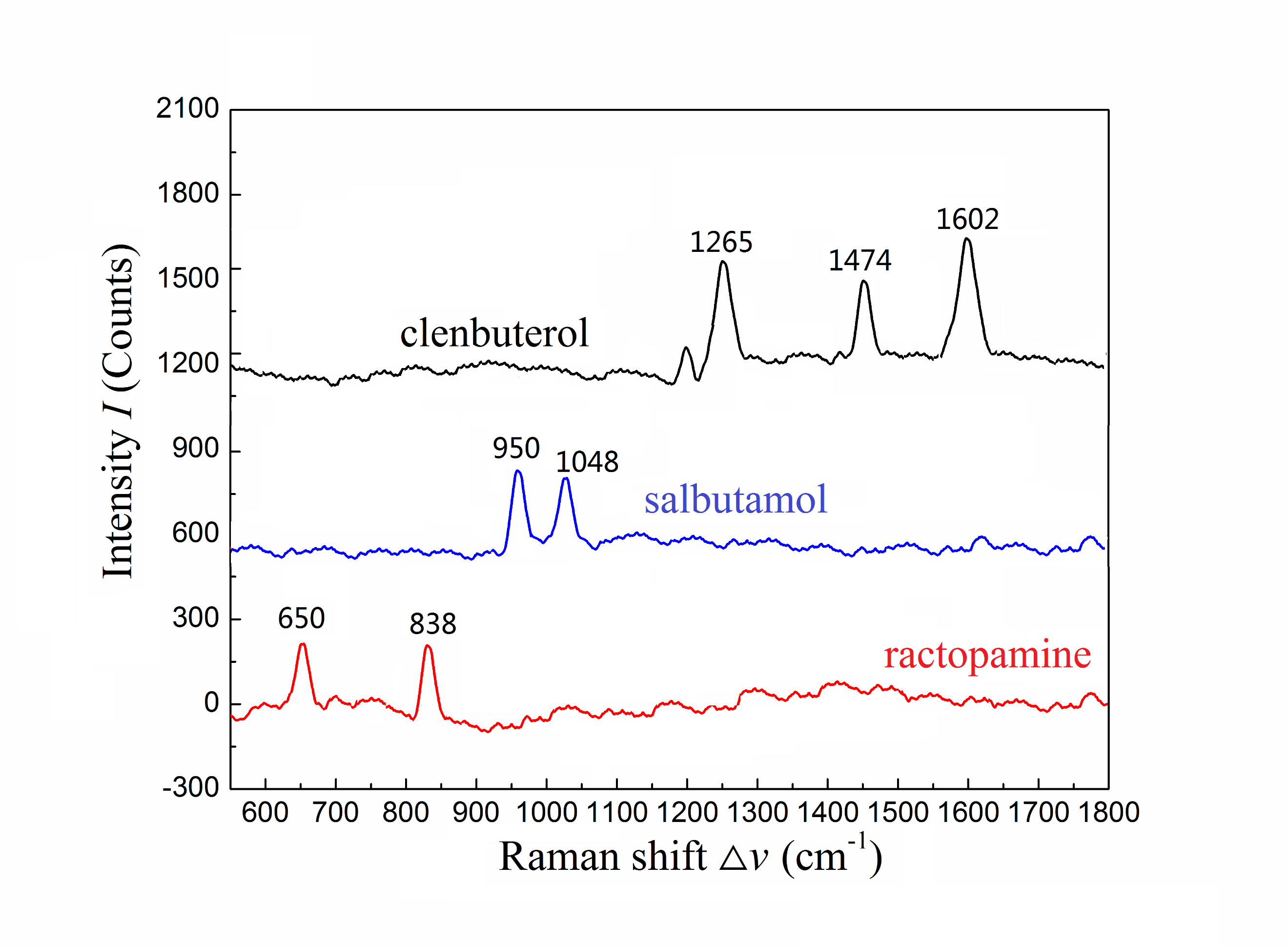
**

**Fig. S5.** SERS spectra of three agonists standard solutions (*C* = 10 ng•mL^−1^)


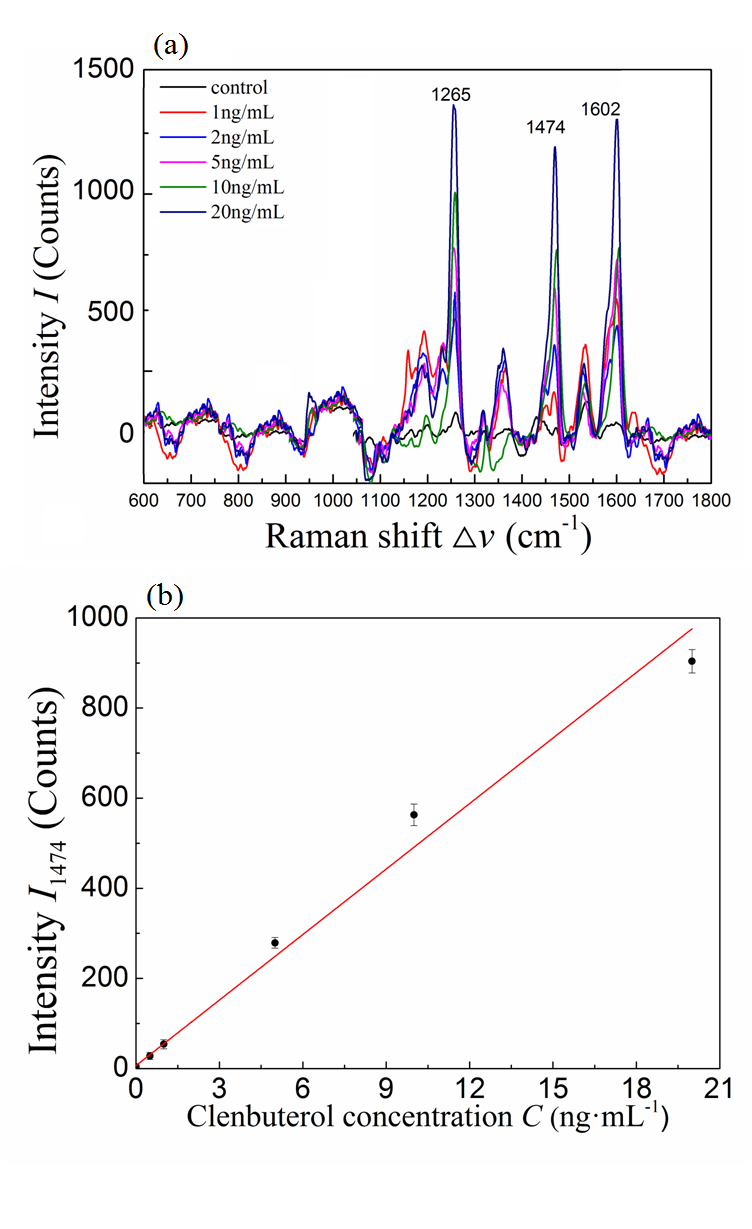


**Fig. S6** (a) SERS spectra of the extracted clenbuterol inoculated urine samples at the present of ractopamine and salbutamol; (b) The plot of *I*_1474_ versus the inoculated clenbuterol concentration *C*.

Table S1. The characteristic peaks of three β_2_-adrenergic agonist.

| characteristic peaks  Δ*v* (cm^-1^) | 650 | 838 | 950 | 1048 | 1265 | 1474 | 1602 |
| --- | --- | --- | --- | --- | --- | --- | --- |
| clenbuterol |  |  |  |  | √ | √ | √ |
| ractopamine | √ | √ |  |  |  |  |  |
| salbutamol |  |  | √ | √ |  |  |  |

**References:**

[1] Ratna T, Brown RJC, Milton MJT (2007) Strategy to improve the reproducibility of colloidal SERS. J Raman Spectrosc 38: 1469-1479.

[2] Princeton University 2014, Section 10: Chemical Specific Information Piranha Solutions. Available online: http://web.princeton.edu/sites/ehs/labsafetymanual/cheminfo/piranha.htm (accessed on 9 April 2007).
